# Public perspectives on disinvestments in drug funding: results from a Canadian deliberative public engagement event on cancer drugs

**DOI:** 10.1186/s12889-019-7303-2

**Published:** 2019-07-22

**Authors:** Sarah Costa, Colene Bentley, Dean A. Regier, Helen McTaggart-Cowan, Craig Mitton, Michael M. Burgess, Stuart J. Peacock

**Affiliations:** 1 Canadian Centre for Applied Research in Cancer Control, 675 West 10th Avenue, Vancouver, British, Columbia V5Z 1L3 Canada; 2 Cancer Control Research, BC Cancer, 675 West 10th Avenue, Vancouver, British Columbia V5Z 1L3 Canada; 3 School of Population and Public Health, University of British Columbia, 2206 East Mall, Vancouver, British Columbia V6T 1Z3 Canada; 4 Centre for Clinical Epidemiology and Evaluation, 828 West 10th Avenue, Vancouver, British Columbia V5Z 1M9 Canada; 5 W. Maurice Young Centre for Applied Ethics, School of Population and Public Health, Southern Medical Program, University of British Columbia, 239 RHS, 1088 Discovery Avenue, Kelowna, British Columbia V1V 1V7 Canada; 6 Faculty of Health Sciences, Simon Fraser University, Blusson Hall, Room 11300, 8888 University Drive, Burnaby, British Columbia V5A 1S6 Canada

**Keywords:** Priority setting, Cancer drug funding, Disinvestment, Public engagement

## Abstract

**Background:**

Decisions relating to the funding of new drugs are becoming increasingly challenging due to a combination of aging populations, rapidly increasing list prices, and greater numbers of drug-indication pairs being brought to market. This is especially true in cancer, where rapid list price inflation is coupled with steeply rising numbers of incident cancer cases. Within a publicly funded health care system, there is increasing recognition that resource allocation decisions should consider the reassessment of, and potential disinvestment from, currently funded interventions alongside new investments. Public input into the decision-making process can help legitimize the outcomes and ensure priority-setting processes are aligned with public priorities.

**Methods:**

In September 2014, a public deliberation event was held in Vancouver, Canada, to obtain public input on the topic of cancer drug funding. Twenty-four members of the general public were tasked with making collective recommendations for policy-makers about the principles that should guide funding decisions for cancer drugs in the province of British Columbia. Deliberative questions and decision aids were used to elicit individuals’ willingness to make trade-offs between expenditures and health outcomes.

**Results:**

Participants discussed the implications of disinvestment decisions from cancer drugs in terms of its impact on patient choice, fairness and quality of life. Their discussions indicate that in order for a decision to disinvest from currently-funded cancer drugs to be acceptable, it must align with three main principles: the decision must be accompanied by significant gains, described both in terms of cost savings and opportunities to re-invest elsewhere in the health care system; those who are currently prescribed a cancer drug should be allowed to continue their course of treatment (referred to as a continuance clause, or “grandfathering” approach); and it must consider how access to care for specialized populations is impacted.

**Conclusions:**

The results from this deliberation event provide insight into what is acceptable to British Columbians with respect to disinvestment decisions for cancer drugs. These recommendations can be considered within wider health system decision-making frameworks for funding decisions relating to all drugs, as well as for cancer drugs.

## Background

Decisions relating to drug funding have become increasingly challenging and complex in the last decade. This is in part due to an ageing population, rapid increases in list prices, and steep growth in the number of drug-indication pairs being brought to market, in particular in cancer [1–3]. In British Columbia (BC), Canada, the cost of take-home cancer medications rose by 134% between 2006 and 2013, and cancer incidence rates are projected to rise by an estimated 40% between 2017 and 2030, predominately among those aged 70 years and older [4, 5]. Despite the fact that new cancer drugs are being approved for use, assessments of their health gains have revealed limited evidence of significant gains in terms of survival or improved quality of life, instead demonstrating that new cancer drugs offer only marginal health benefits for their cost [1–3, 6–8]. The growth in cancer drug expenditure coupled with concerns over the value of new cancer drugs in terms of their costs and benefits have prompted decision-makers to consider the re-assessment – and potential disinvestment from –“low-value” drugs [9]. The rising cost of cancer drugs, coupled with modest improvements in health benefit and increasing cancer incidence rates, raises concerns about health system affordability, and places the issue at the forefront of health policy decision-making.

Decision-makers responsible for health system budgets are tasked with making difficult decisions about how to allocate limited health-care resources within budget constraints. Economic evaluations of health-care technologies can help by providing cost-effectiveness evidence. Economic evidence is a key input into Priority Setting and Resource Allocation (PSRA) decision-making frameworks [10]. PSRA frameworks are used by decision-makers to make decisions about where health-care resources would best be spent in order to maximize health-care system goals, including both investments and disinvestments in health-care interventions [11]. In Canada, new cancer drug indications are reviewed by the pan-Canadian Oncology Drug Review (pCODR) at the Canadian Agency for Drugs and Technologies in Health (CADTH), which makes funding recommendations for the provincial and territorial public drug plans^1^ based on four factors: clinical effectiveness; cost of the drug; patient values and preferences; and feasibility of integration into the health care system [12]. The final drug price for each province and territory is negotiated by pan-Canadian Pharmaceutical Alliance (pCPA). Provincial and territorial ministries of health then make jurisdictional decisions about whether or not to list the drug based on pCODR’s recommendations and the price negotiated by pCPA. Processes for identifying drugs eligible for disinvestment from the drug formulary in Canada are not as well established, despite disinvestment being a critical part of the priority-setting process [13].

Disinvestment has been defined as, “the process of (partially or completely) withdrawing health resources from any existing health care practices, procedures, technologies or pharmaceuticals that are deemed to deliver little or no health gain for their cost”, and is challenging to undertake in practice [14, 15]. There may be several reasons for disinvestment, including economic (e.g., maximize efficiency), organizational (e.g., ensure sustainability of the system), and social (e.g. responsible spending of public funds). While investments in health care are often accompanied by funding and/or reimbursement structures intended to help incentivize their uptake, disinvestments may be associated with the removal of available options or be seen as a ‘cost-cutting’ strategy. This can propagate a desire to maintain status quo, which could include deferring approval of new drugs to accommodate existing drug funding arrangements [16–18]. Moreover, decisions to de-list health care services can incite backlash from clinicians, patients or other consumers, making it a challenging process especially in cancer amidst often strong personal and emotional associations with the disease [19–21]. A recent review of 40 different countries’ experiences with disinvestment found evidence of fifteen programs from eight countries reported in the literature, including Australia, the UK, the USA’s Choosing Wisely campaign, and the application of Programme Budgeting and Marginal Analysis (PBMA) and evidence-based PSRA analyses in Canada. Possible reasons for the lack of disinvestment strategies in drugs include limited appetite to discuss disinvestment in the literature and resistance to changing prescribing behaviours in practice [9].

Negative associations with disinvestment could be shared by members of the public, making it difficult to accept the fact that there is a need to disinvest from ‘low-value’ cancer drugs [22, 23]. This has prompted some HTA agencies to demand transparency in disinvestment practices and the frameworks that guide them, in particular to make explicit the need for public input in addition to other relevant stakeholders [24–26]. Public engagement regarding disinvestment decisions has been sought in the areas of assisted reproductive technologies, vitamin B_12_/folate pathology testing, beta interferons for multiple sclerosis, and decommissioning of older people’s care home services, among others [18, 20, 27–30]. However, little is known about public values and perspectives regarding disinvestment from drugs, and more specifically, cancer drugs. Although there is strong support from Canadian decision-makers to use public and patient input to inform priority-setting, few have actively incorporated these data into their decision-making processes [31]. Additionally, the methods used to elicit public opinion on contentious topics have predominately been consultative (e.g., focus groups); however, these approaches are limited in their ability to explore the complex social factors and incentives embedded in public attitudes [29, 32, 33]. Deliberative methods instead use an iterative process that relies on the respectful exchanging of views and opinions to address complex public policy problems [34–37]. Unlike other methods of engagement, participants collectively establish and present recommendations within a deliberative public engagement without filtering of the data. Considering that disinvestment can be a contentious topic in health care, its application to cancer drugs is a suitable subject for public deliberation.

We held a public deliberative engagement event in BC, Canada to understand what is important to British Columbians regarding cancer drug funding. Over two non-consecutive weekends in September 2014, 24 participants from across BC deliberated and discussed policy-relevant issues, including disinvestment, acceptable trade-offs for cancer drugs, and governance in decision-making processes. A high-level overview of the recommendations and disagreement from that event has been published elsewhere [38]. The purpose of this paper is to report on the findings specifically regarding the topic of disinvestment from cancer drugs. This study adds to the evidence base regarding public involvement in disinvestment decisions by identifying the conditions that this group of participants felt is necessary in order to accept a decision to disinvest from currently-funded cancer drugs.

## Methods

### Recruitment

Recruitment of participants for the deliberation is described in Bentley et al. [38]. Briefly, the goal of recruitment was to ensure diversity of perspectives and preferences among participants. They were recruited to ensure representativeness against key demographic characteristics of the 2006 BC census data including experience with chronic disease, parenthood, rurality, and income and education. Exclusion criteria were: being employed by, or having a direct financial relationship with, a tobacco company; currently participating in lobbying for a health advocacy group; employed as a health policy maker; and not available to attend both weekends. A market research company was used to recruit participants. Thirty participants were selected to participate in the deliberative event using an algorithm that ensured representativeness of demographic characteristics—serving as a proxy for lived experience—as well as preferences elicited through administration of a Discrete Choice Experiment. In total, 24 participants attended the event (see Table 1 [38]). This study was approved by the University of British Columbia – British Columbia Cancer Research Ethics Board (#H11–02226). All participants signed a consent form before participating in the deliberation event.

**Table 1:** Participant characteristics

	No. of participants (*N* = 24)	Percent (%) of total
Sex		
Female	13	54.2
Male	11	45.8
Age		
18–24	1	4.2
25–34	5	20.8
35–49	4	16.7
50–64	9	37.5
65+	5	20.8
Regional Health Authority^a^ of primary residence		
Vancouver	8	33.3
Fraser	7	29.2
Island	4	16.7
Interior	4	16.7
Northern	1	4.2
Experience with a chronic illness (personal or caregiver)		
No	16	66.7
Yes	8	33.3
Ethnicity		
Caucasian	16	66.7
Chinese	3	12.5
South Asian	1	4.2
Aboriginal	1	4.2
Other	3	12.5
Highest level of education attained		
High school	6	25.0
Some university	2	8.3
University or College	16	66.7
Annual household income		
< $20,000	3	12.5
$20,000 – $34,999	3	12.5
$35,000 – $49,999	3	12.5
$50,000 – $79,999	7	29.2
$80,000+	8	33.3
Have children?		
Yes	13	54.2
No	11	45.8

^a^A map of the Regional Health Authorities can be found here: https://www2.gov.bc.ca/gov/content/health/about-bc-s-health-care-system/partners/health-authorities/regional-health-authorities

### Structure of the event

The general structure and format of the deliberative event was based on the methods developed by Burgess and O’Doherty, and is explained further in Bentley et al. [38, 39]. The event spanned two non-consecutive weekends, with the first weekend dedicated to an introduction of the discussion topics in both small- and large-group formats, and the second weekend focused on making collective recommendations to policy makers on the event topics and voting on them. Any points of contention or disagreement with the recommendations were captured. Invited speakers presented a range of perspectives, including cancer patients, cancer advocacy groups, oncologists, rural and indigenous physicians, decision-makers and health economists. Speakers and decision-makers were present on the last day of the event to hear participants make their recommendations and describe their reasoning behind them.

Two methods were used to explore participants’ views on disinvestment (see 1 and 2 below, respectively): (1) a deliberative question that asked participants to consider the conditions under which they would accept a decision to disinvest as acceptable (day 2); and (2) a decision scenario involving a trade-off between cost savings and health benefits (day 3). Participants discussed the deliberative question in small-group and large-group formats, made recommendations based on those discussions and voted on them. Trained facilitators encouraged discussion among participants.

1. Under what conditions is there an obligation to continue to fund a cancer drug when new information suggests the drug is not as desirable as previously determined?

(2) As a decision-maker, consider trade-offs regarding the appropriateness of continuing to fund a drug with differences in cost, quality of life, and length of life, in order to determine at what cost to the total budget funding of the current drug should be discontinued.

**Fig. 1:**
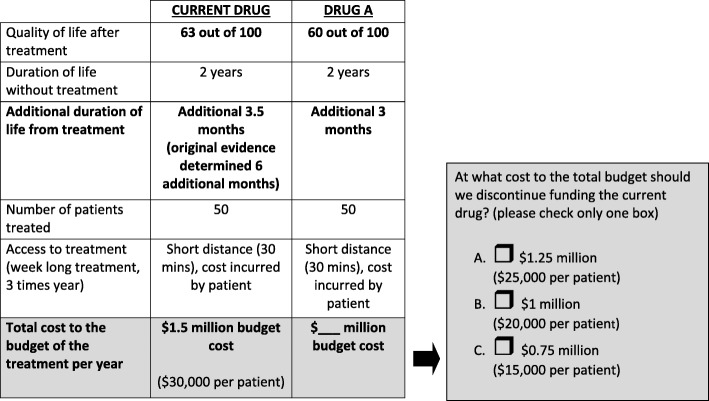
Decision scenario on disinvestment. The decision scenario was used as a thought exercise to encourage consideration of what trade-offs need to be made in order for a new treatment option, drug A, to be accepted in place of the current drug. Participants were asked, “at what cost to the total health care budget should we discontinue funding the current drug?”

The decision scenario presented participants with an option between two hypothetical drugs: a currently funded drug, and a new drug with a lower cost (Fig. 1). Drug classification and cancer type, as well as the nature of the treatment were purposefully unspecified to avoid the formation of pre-conceived attitudes or judgements about the disease or affected population. The scenario included characteristics that differed between the drugs, including differences in quality of life, duration of life after treatment and cost. Participants were asked to deliberate on what the cost of the new drug would have to be in order to justify a switch from the current drug to the new drug. The options were: a difference of 5,000 per patient (option A) 10,000 (option B), or $15,000 (option C). The expressed trade-off for participants was a loss of quality of life (3 points on a scale of 0–100) and loss of duration of life after treatment (difference of 0.5 months), for the opportunity of cost savings that could be reinvested elsewhere in the health care system.

For each of the deliberative questions and decision scenarios used in the event, participants were asked to assume the role of a decision-maker. During their deliberations, participants were encouraged to consider the scenarios and questions from a collective point of view and make recommendations that were socially acceptable to the group. The facilitators encouraged participants to provide reasoning for their choices, which were then explored further in the large-group setting.

### Transcript analysis

Analysis of the transcripts from the event was done using NVivo qualitative data analysis software (QSR International Pty Ltd., Version 10, 2012). All sessions were coded and analyzed by one primary reviewer (CB), with a second reviewer (SC) independently coding a portion of the transcripts for quality control. A primary analyst (SC) coded specifically on the topic of disinvestment. Participants were de-identified and given a pseudonym for transcript analysis and reporting of results.

## Results

The deliberative question and decision scenario were used to prompt participants to think about some of the trade-offs and factors that were important to them when considering disinvesting from currently funded cancer drugs. Through small- and large-group discussions, participants demonstrated their understanding of the tasks, and considered their roles as decision-makers and good stewards of public funds seriously (“Because it is, after all, public funding you’re working with” - Participant 13, Small group G, Day 2). Issues around access to care, patient choice, the impact that new information should have on disinvestment decisions, and opportunity cost were discussed.

### Access to care

Participants were concerned that disinvestment decisions would create disparities between patients based on place of residence. Discussions around disinvesting from currently funded drugs were closely tied to the notion of creating barriers to access, especially for drugs taken by intravenous versus oral drug administration. In formulating their recommendations around access to care, participants wanted to ensure that a decision to disinvest did not disadvantage people in “rural communities” (Participant 1, Large Group Day 2) and other populations with access challenges:

PARTICIPANT 4: We can’t just pick, “[---] It’s good for us but forget you guys [---] because you guys moved up north”. You made a commitment to the patient, the drug’s been approved, the doctors began giving it. Where is our moral obligation to the patient?

(Small group G, Day 2)

Additionally, some participants felt it was important that access to the drug included more than geographical considerations, extending to other marginalized populations (“sub-groups”) who might be adversely affected or disadvantaged by policy decisions:

PARTICIPANT 7: I am thinking about other sub-groups, like maybe people with limited mental capacity, or street people, other vulnerable populations like that. Do they fit? (Large group, Day 2)

Participants formulated a recommendation that combined access to care issues that were based on geographical considerations as well as “vulnerable” populations. The recommendation was: “There is an obligation to continue to fund a cancer drug if discontinued funding would have a negative impact on populations in rural communities and others with limited access.” All participants agreed with this recommendation.

### Patient choice

In their discussions, participants voiced concerns about how a decision to disinvest would disrupt the patient’s care experience because it may introduce side effects and tolerance issues that patients would want to avoid. As described by one participant:

PARTICIPANT 6: I think it’s just, like, when you’re sick and stuff like that, and you’re on a certain drug, that being switched from one drug to another drug [---] is just disturbing.

(Small group Y, Day 3)

Participants who had experience as a patient themselves, as well as many other participants, described a desire to avoid disrupting the stable state of being accustomed to the current medication and its side-effects. Some wanted to avoid the anxiety and fear that goes along with changing drugs:

PARTICIPANT 11: Well, any time you switch to a new medication you have an opportunity for new side effects. And that’s the thing, your body may get used to a medication, it may be effective and you may have weeded out those initial side effects that you had. [---]Any time you take something new, you open that window again. (Small group B, Day 2)

PARTICIPANT 10: Psychologically, people feel comfortable with what they’re taking and there’s this little bit of fear changing to something new. Maybe it won't work as well, even though it’s cheaper. So I think psychologically for a patient that’s doing well on that particular drug, to move them [to a different drug] could have terrible ramifications. (Large group, Day 4)

Participants wanted assurance that a patient would be allowed to continue on the currently funded drug until their course of treatment was finished. This concept was expressed as preserving a patient’s choice to remain on the current drug if it “works for them” (Participant 1, Small group Y, Day 2). Participants described this as a “grandfather clause” or a continuance clause:

PARTICIPANT 4: I think we covered that yesterday with the grandfather clause, didn't we?

VOICES: Yeah.

PARTICIPANT 4: If the patient is on [the soon-to-be delisted drug] they would be under the grandfather clause. [---] We started them on this drug, so we kind of had to give them the choice to finish it. (Large group, Day 4)

The recommendation that the group voted on was: “Patients who are taking an existing drug should have the option to stay on the existing drug even if it is more expensive than a similar new drug.” All participants agreed with this recommendation.

The concept of patient choice also appeared in a subsequent recommendation: “There is an obligation to continue to fund a cancer drug if it is significantly easier to use compared to other drugs or treatments (for example, oral vs. intravenous drugs).” Most participants agreed with this recommendation, but concerns were raised that ease of use was not sufficient enough to warrant ongoing funding of a drug:

PARTICIPANT 4: So yes, this drug is easier to use, [---] but there is an alternative drug. It’s harder to use, but the benefits are more. Then, do we continue funding just because it is easier to use? (Large group, Day 2)

Some participants, like Participant 4, described a need for multiple criteria to be considered in disinvestment policy decisions, such as overall effectiveness of the drug and its side effects in providing more health benefit.

### New information

Participants generally supported a decision to re-evaluate drugs when new evidence about that drug’s effectiveness becomes available. They did, however, discuss the fact that some element of subjectivity would factor into a decision to determine whether that new information was significant enough to warrant a change in clinical practice. For example, some debated what was meant by “significant” and what the implications to patients would be before making a decision to disinvest or not. As described by Participants 7 and 6 below:

PARTICIPANT 7: And I also think though that another really important point is that [switching to a new drug] depends on [---] the degree of the loss in desirability. So, like what I’m saying, how much more undesirable is it?

FACILITATOR: How much more undesirable is it?

PARTICIPANT 7: Yeah. Like is it causing people to die? Is it causing people to get sleepy? Like, how significant is this new information. (Small group Y, Day 2)

PARTICIPANT 6: I just wanted to say, I think that it's important that you not be changing the drugs too often [---] you know, there's way to determining [*sic*] whether or not the evidence is significant. And if you were to change every time some evidence suggested [you should] but the evidence wasn't very strong, I mean you'd burn up a lot of time just switching drugs all the time. (Large group, Day 4)

Participants were also concerned about who determines the evidence to be significant. Some recommended that an independent arms-length body should be evaluating the information and making recommendations. This was some of the reasoning behind another recommendation participants made: “There is an obligation to continue to fund a cancer drug when the new scientific information/evidence that suggests the drug is not as desirable as previously determined is not significant or conclusive.”

### Trade-offs

When considering the disinvestment scenario and having to make a trade-off between a currently-funded drug and a new drug (Fig. 1), some participants were reluctant to switch, citing concerns about the need to rebuild tolerance to the new drug’s side effects. For others, a decision to disinvest meant giving something up that was important to participants, including decreased quality of life or length of life:

PARTICIPANT 13: When you're under treatment for something you’ve been approved already. You’re comfortable with it.

(Small group G, Day 3)

PARTICIPANT 5: I would be really ticked off if I found out there is another drug out there that was better. More expensive than -- “Here, have this cheap stuff.”

(Small group B, Day 3)

The information provided in the disinvestment scenario was used to frame how participants thought about trade-offs when faced with a decision to discontinue funding for the current drug. For example, some calculated what the maximum cost savings was (Fig. 1, option C) given the scenario options, and then used that information to discuss how those savings could be used to fund other things in the health care system. Participants debated the significance of cost savings against what would be lost with the switch to a new drug (i.e., quality of life):

PARTICIPANT 11: If we can get [the drug] for say half the price, [---] then we have $750,000 to spend on another drug or to spend on more drugs to help more people. That's the trade-off I see.

PARTICIPANT 8: Three points out of a hundred [difference on the quality of life scale] is like barely even noticeable. [---] They're almost the same.

PARTICIPANT 17: I am starting to agree with [Participant 11] because it is true that there would be money, I mean, for other areas, right?

PARTICIPANT 5: Well, ketchup is ketchup, but nothing beats Heinz.

PARTICIPANT 11: But if you bought cheaper ketchup, you can afford some mustard too.

(Small group B, Day 3)

The expectation of maximum cost savings in order to disinvest was common (Fig. 1, option C):

FACILITATOR: Would saving $5,000 per patient be enough?

PARTICIPANT 4: My immediate response would be no.

PARTICIPANT 18: It’s not really good enough –.

FACILITATOR: What about $10,000 per patient?

PARTICIPANT 13: No, we want $15,000.

(Small group G, Day 3)

For some, the loss in health benefit represented too significant a compromise to switch to the new drug, despite the potential for cost savings. An alternative option was proposed instead, one in which the status quo would be maintained and the current drug would remain funded:

PARTICIPANT 10: We want a “D” option. Zero, do not discontinue.

PARTICIPANT 5: I wouldn’t switch. [---] I can't agree with Drug A because the quality of life has gone down.

(Small group B, Day 3)

PARTICIPANT 13: [C]an we have that option D? Don't do it at all? [---]

(Small group G, Day 3)

## Discussion

Our deliberative public engagement has provided an opportunity to understand the factors important to this group of British Columbians regarding cancer drug funding decisions. The results described in this paper capture participants’ recommendations and reasons for them when considering disinvestments in cancer drugs.

Overall, participants were keen to contribute to the event and work collectively to come up with solutions to the complex policy issues presented to them [38]. Their discussions regarding acceptable trade-offs within the context of disinvestment decisions demonstrated a capacity to discuss complex policy topics while drawing on their own experiences, as well as knowledge gained throughout the event. Our work provides supporting evidence that a diverse public can be engaged to inform problem-solving around challenging policy topics [25, 40, 41]. Participants understood the principle of setting limits and the notion that difficult funding decisions need to be made [38].

Our analysis revealed that in order for a decision to disinvest to be considered acceptable to this mini-public, it must align with three main principles. First, the decision must demonstrate significant gains, such as cost savings. For those participants who were motivated by cost, they demanded considerable savings in order to justify a switch from the current to the new drug. Generally however, most participants did not approach decision-making ‘at the margin’. In other words, the marginal benefit of a decision to disinvest from a cancer drug in terms of the opportunities for re-investment elsewhere in the health care system, was not enough of a motivator to be considered an acceptable choice. Instead, the focus of the disinvestment decision was often framed in terms of what would be lost – namely, quality of life (“I can’t agree with Drug A because the quality of life has gone down” – Participant 5) and familiarity with and tolerance of side effects on currently-funded drugs. Participants did not want to have to settle for an inferior drug or make compromises on quality of life for cost savings. Participants’ desire to avoid a change in cancer drugs when it came to disinvestment decisions could have implications for other types of drug treatments, for example, biosimilar oncology drugs. Biosimilars are highly similar copies of originator biological drugs that can be made at a lower cost [42, 43]. According to FDA guidelines, a biosimilar that is found to be highly similar to, and interchangeable with, the reference product can be used as a direct substitute [44]. Patient acceptability of biosimilars is important to ensure uptake of these products [45, 46]. A recent study of patient attitudes toward biosimilars in the context of rheumatoid arthritis found that patients had some reluctance to being switched to a biosimilar, with some expressing concerns of being offered a “cheaper and less-effective” version of the drug [45]. In our deliberations, participants voiced concerns with being switched to a different – albeit similar – drug in terms of what it could mean for the patient psychologically: “So I think psychologically for a patient that’s doing well on that particular drug, to move them [to a different drug] could have terrible ramifications” (Participant 10). This may have implications for preferences toward the use of biosimilars. To offset the loss, participants proposed instituting a continuance – or so-called grandfather – clause as part of their recommendations to policy-makers. This second principle was supported by all participants [47]. The continuance clause would allow patients currently being treated to finish their course of treatment without being switched to a different drug. This principle was rooted in respect for patient choice and fairness (as one participant explained, instituting a continuance clause was “giving [patients] the right to choose” – Participant 4, Large group Day 4), and a desire to preserve good quality of life unaffected by new side effects or tolerance issues from switching drugs. In their deliberations, participants made recommendations for how the continuance clause could be implemented in practice, suggesting a gradual phasing out of the currently used drug allowing current patients to finish their course of treatment while patients starting a new treatment regime would only be prescribed the new drug.

Finally, the third principle supported by participants was that a decision to disinvest must consider how that decision would affect specific populations in society (“those with limited mental [health] capacity, or street people, other vulnerable populations like that”). BC is a province with over four million residents with more than 14% of the population living in rural settings [48]. Improving access to care for rural and/or remote geographic areas has been well described as a priority for policy makers [49]. The public is keenly aware of issues of access that affect citizens, with media attention often being drawn to these issues, in particular related to health care. Participants wanted to ensure that specific populations would not be disadvantaged by a decision to disinvest. This reasoning was strongly tied to discussions around access to oral versus intravenous drug administration, with many participants supporting the notion of improving access to oral medications to reduce potential barriers to care.

This study adds to the evidence base on the topic of public involvement in disinvestment decisions by identifying the conditions under which members of the general BC public would be willing to accept a decision to disinvest from currently-funded cancer drugs. Proponents of technology assessment and reassessment decisions in health care recognize the need for public involvement in these decisions to ensure they are transparent and aligned with public values [15, 50]. In BC, decision making committees can look to the results from this study for practical guidance on how to undertake disinvestment of cancer drugs. For example, the notion of a continuance clause supports the recommendation for a sunset clause made by Canada’s HTA agency, CADTH, over a decade ago as a policy approach to disinvestment of obsolete health care technologies [15]. Mode of drug administration – oral versus intravenous – could also be considered as criteria in any disinvestment or reassessment framework. The results of this study should be taken in context with the fact that there is no agreed-upon framework for disinvestment [51]. Regardless, decision making frameworks would benefit from the incorporation of what members of the BC public consider important to a disinvestment decision – that is, demonstration of significant gains, institution of a continuance clause and regard for the protection of historically disadvantaged populations – in order to engage the public in the process and develop a common appreciation for reassessment of cancer drugs.

Our study has the following limitations. As is the case with all qualitative research, results are highly context-specific; here, they reflect a particular public’s perspective on drug disinvestment decisions. Caution must be taken in generalizing the results to other settings or policy questions. Our event was specific to this group of participants from BC, and cannot necessarily be generalized to other settings. The impact of the expert speakers’ perspectives has various degrees of influence on the participants’ deliberations. Speakers shared perspectives about rural cancer care and being a cancer patient, and many participants referred to these topics in their discussions. Decision scenarios were also constructed to guide participants’ thinking around specific trade-offs; however, this had the effect of limiting the range of topics discussed and recommendations produced.

## Conclusion

The findings from our public deliberation event have shown that members of the general public can come together in a deliberative engagement format to make recommendations on complex issues such as cancer drug funding. Our findings suggest that in order for a decision to disinvest from a cancer drug to be acceptable to members of the general public, it must demonstrate significant value in the form of cost savings without reducing quality of life; be concerned with how the decision impacts disadvantaged populations and those living in rural/remote settings; and be accompanied by a plan for gradual phasing-out of the currently funded drug that protects patients’ right to choose how they wish to finish their course of treatment. It is most instructive that the participants did not shy away from disinvestment, but sought to provide guidance about the conditions under which they would consider disinvestment justified and fair. Disinvestment considerations are highly contextualized to the population(s) affected and the drug being displaced. The public values sought in this deliberation event provide a set of principles for approaching disinvestment policy making that can be incorporated into broader priority-setting frameworks in health care.

## Data Availability

The datasets generated and/or analysed during the current study are not publicly available in order to protect the confidentiality of participants. De-identified data may be available from the corresponding author on reasonable request.

## Abbreviations

ARCC: Canadian Centre for Applied Research in Cancer Control
CADTH: Canadian Agency for Drugs and Technologies in Health
CIHR: Canadian Institutes of Health Research
INESS: Institut national d’excellence en santé et en services sociaux
PBMA: Programme Budgeting and Marginal Analysis
pCODR: pan-Canadian Oncology Drug Review
pCPA: pan-Canadian Pharmaceutical Alliance
PSRA: Priority Setting and Resource Allocation

## Footnotes

1 Excluding Quebec, which follows a similar process under the Institut national d’excellence en santé et en services sociaux (INESS)
